# A polarized multicomponent foundation upholds ciliary central microtubules

**DOI:** 10.1093/jmcb/mjae031

**Published:** 2024-08-20

**Authors:** Qingxia Chen, Huijie Zhao, Xinwen Pan, Chuyu Fang, Benhua Qiu, Jingting Guo, Xiumin Yan, Xueliang Zhu

**Affiliations:** Ministry of Education–Shanghai Key Laboratory of Children's Environmental Health, Institute of Early Life Health, Xinhua Hospital, Shanghai Jiao Tong University School of Medicine, Shanghai 200092, China; State Key Laboratory of Cell Biology, Shanghai Institute of Biochemistry and Cell Biology, Center for Excellence in Molecular Cell Science, Chinese Academy of Sciences, Shanghai 200031, China; Institute of Biomedical Sciences, College of Life Sciences, Key Laboratory of Animal Resistance Biology of Shandong Province, Collaborative Innovation Center of Cell Biology in Universities of Shandong, Shandong Normal University, Jinan 250014, China; School of Life Science and Technology, ShanghaiTech University, Shanghai 201210, China; Center for Excellence in Molecular Cell Science, University of Chinese Academy of Sciences, Beijing 100049, China; State Key Laboratory of Cell Biology, Shanghai Institute of Biochemistry and Cell Biology, Center for Excellence in Molecular Cell Science, Chinese Academy of Sciences, Shanghai 200031, China; Center for Excellence in Molecular Cell Science, University of Chinese Academy of Sciences, Beijing 100049, China; State Key Laboratory of Cell Biology, Shanghai Institute of Biochemistry and Cell Biology, Center for Excellence in Molecular Cell Science, Chinese Academy of Sciences, Shanghai 200031, China; Center for Excellence in Molecular Cell Science, University of Chinese Academy of Sciences, Beijing 100049, China; School of Life Science and Technology, ShanghaiTech University, Shanghai 201210, China; Center for Excellence in Molecular Cell Science, University of Chinese Academy of Sciences, Beijing 100049, China; Ministry of Education–Shanghai Key Laboratory of Children's Environmental Health, Institute of Early Life Health, Xinhua Hospital, Shanghai Jiao Tong University School of Medicine, Shanghai 200092, China; State Key Laboratory of Cell Biology, Shanghai Institute of Biochemistry and Cell Biology, Center for Excellence in Molecular Cell Science, Chinese Academy of Sciences, Shanghai 200031, China; School of Life Science and Technology, ShanghaiTech University, Shanghai 201210, China; Center for Excellence in Molecular Cell Science, University of Chinese Academy of Sciences, Beijing 100049, China; School of Life Science, Hangzhou Institute for Advanced Study, University of Chinese Academy of Sciences, Hangzhou 310024, China

**Keywords:** basal plate, central pair, ciliary motility, ependyma, hydrocephalus, motile cilia

## Abstract

Cilia's back-and-forth beat pattern requires a central pair (CP) of microtubules. However, the mechanism by which the CP is upheld above the transition zone (TZ) remains unclear. Here, we showed that a rod-like substructure marked by Cep131 and ciliary Centrin serves as a polarized CP-supporting foundation. This CP-foundation (CPF) was assembled independently of the CP during ciliogenesis in mouse ependymal cells. It protruded from the distal end of the basal body out of the TZ to enwrap the proximal end of the CP. Through proximity labeling, we identified 26 potential CPF components, among which Ccdc148 specifically localized at the proximal region of Centrin-decorated CPF and was complementary to the Cep131-enriched distal region. *Cep131* deficiency abolished the CPF, resulting in CP penetration into the TZ. Consequently, cilia became prone to ultrastructural abnormality and paralysis, and *Cep131*-deficient mice were susceptible to late-onset hydrocephalus. In addition to *Centrin*, phylogenetic analysis also indicated conservations of *Ccdc131* and *Ccdc148* from protists to mammals, suggesting that the CPF is an evolutionarily conserved multicomponent CP-supporting platform in cilia.

## Introduction

Cilia, hair-like organelles assembled from a specialized centriole or the basal body (BB), exert diverse motile and sensory functions important for organism development and homeostasis (Nigg and Raff, 2009; Bettencourt-Dias et al., 2011; Breslow and Holland, 2019). Primary cilia are usually immotile and consist of nine peripheral microtubule (MT) doublets (‘9 + 0’), whereas most motile cilia additionally contain a central pair (CP) of MTs (‘9 + 2’) (Ishikawa, 2017; Loreng and Smith, 2017; Reiter and Leroux, 2017; Lyu et al., 2024). The two central MTs, designated respectively as C1 and C2, are each coated with unique protein complexes to form the central apparatus (CA). The CA is bridged with the nine peripheral doublets by radial spokes to regulate ciliary beat forms (Loreng and Smith, 2017; Zhao et al., 2020; Liu et al., 2021). Defects in ciliary structure or function cause diverse human diseases known as ciliopathies (Fliegauf et al., 2007; Reiter and Leroux, 2017).

While the axonemal MT doublets are extensions of centriolar MT triplets, the CP consists of two non-centrosomal MTs whose minus ends are usually terminated at the top of the transition zone (TZ) (Loreng and Smith, 2017; Reiter and Leroux, 2017), a ciliary diffusion barrier (Garcia-Gonzalo and Reiter, 2017). Whether the minus ends require a foundation to hold them in position to allow proper functions of the CA in ciliary beat, however, is unclear. In protists, the CP is found to terminate at electron-dense structures of various shapes and names (commonly termed basal plates) at the top region of the TZ (Ringo, 1967; Kennedy and Zimmerman, 1970; Tucker, 1971; Dute and Kung, 1978; Jarvik and Suhan, 1991; Hoog et al., 2014). For instance, the basal plate is usually plate-shaped in *Trypanosoma* (Hoog et al., 2014; Dean et al., 2019) and contains two central cylinders associated with multiple stellate fibers in *Chlamydomonas* (Jarvik and Suhan, 1991; Hoog et al., 2014). The only basal plate-specific component reported thus far is the trypanosome basalin. Depletion of basalin by RNA interference (RNAi) markedly impairs both the basal plate and the CP (Dean et al., 2019), suggesting that the basal plate is required for CP assembly or stability. Nevertheless, basalin is somehow poorly conserved, and its orthologs have only been identified in *Trypanosoma* and *Leishmania* (Dean et al., 2019). On the other hand, Centrin, which is commonly known as a protein distributed in the distal lumen of centrioles or BBs, also localizes to the basal plate in *Chlamydomonas* as revealed by immuno-electron microscopy (Geimer and Melkonian, 2005). RNAi-mediated depletion of Centrin in *Chlamydomonas* disrupts the basal plate and also severely impairs BB docking and flagellar formation (Koblenz et al., 2003). In contrast, the *Chlamydomonas* mutant carrying a Glu-to-Lys mutation in Centrin (*vfl*-2) contains flagella but displays a disrupted basal plate and frequent CP penetration into the BB (Jarvik and Suhan, 1991; Taillon et al., 1992), suggesting a role of the basal plate as a CP-supporting platform.

Mammalian ‘9 + 2’-type motile cilia generally lack an electron-dense structure reminiscent of the basal plate at the CP proximal end region (Arima et al., 1984; Swiderski et al., 2012; Liu et al., 2021). We previously reported that fibrogranular materials (FGMs), large arrays of electron-dense granules in mammalian multiciliated cells (MCCs) (Sorokin, 1968; Anderson and Brenner, 1971; Dirksen, 1971), enrich many centriolar and ciliary components to facilitate their timely assembly during multiciliation (Zhao et al., 2021). Three-dimensional structured illumination microscopy (3D-SIM) revealed that Cep131 (also known as Azi1), a centriolar satellite protein (Staples et al., 2012; Hall et al., 2013) enriched in FGMs, defines a cylindrical structure at the base of the ciliary central lumen. This structure, tentatively referred to as the ‘CP-foot’, elongates following ciliogenesis to up to ∼2 μm in cultured mouse ependymal cells (mEPCs) and appears to support the CA of ependymal multicilia (Zhao et al., 2021). Interestingly, Centrin co-localizes with Cep131 at the CP-foot (Zhao et al., 2021), in addition to its well-documented distribution in the BB (Paoletti et al., 1996; Garcia-Gonzalo and Reiter, 2017; Laporte et al., 2022), implying a possible correlation between the CP-foot and the basal plate.

In this study, we demonstrate that the CP-foot is a multi-domain substructure that holds the CP in position by enwrapping the CP minus-end region. To better reflect its function and also avoid potential confusion with the term ‘basal foot’ (Nguyen et al., 2020), we hereby rename it the ‘CP-foundation’ (CPF). Furthermore, taxonomic analyses of its components suggest its full conservation from mammals down to choanoflagellates, the closest known protozoan relatives of Metazoa (King et al., 2008; Pinskey et al., 2022), but only partially conserved in the majority of protists, implying a remote relationship between mammalian CPF and protist basal plates.

## Results

### The CPF enwraps the proximal end of the CP

**Figure 1:**
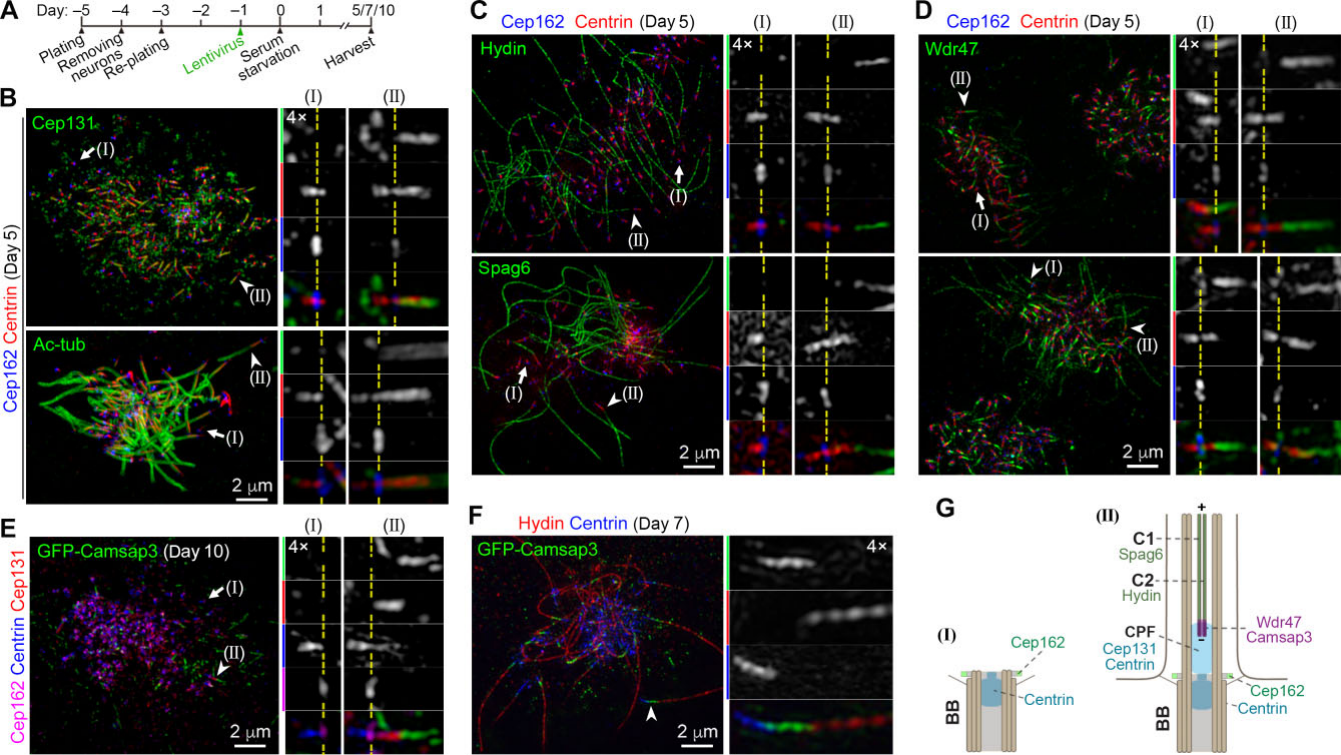
The CP proximal end is embedded into the CPF. (**A**) Experimental scheme. Radial glia were cultured as illustrated for imaging. Lentiviral infections were performed to express GFP-Camsap3. (**B**) Localization of the CPF in ependymal cilia. Cep131 and Centrin served as CPF markers. Cep162 and Ac-tub marked the centriolar distal end and the axoneme, respectively. Representative BBs lacking (arrows) or containing (arrowheads) the CPF are shown details in magnified insets. Dashed lines indicate the position of the Cep162-marked centriolar distal end. (**C**) Relationship between the CA and the CPF. Spag6 and Hydin served as markers for the CA. (**D** and **E**) Relationship between the CP proximal end and the CPF. Wdr47 (**D**) or GFP-Camsap3 (**E**) was used to visualize the CP proximal end. Note that BBs lacking a CPF are also devoid of the CP (arrows). (**F**) Camsap3-decorated CP proximal end region largely lacks Hydin. BBs in magnified insets in **B**–**F** are presented with their proximal-to-distal orientations from left to right. (**G**) Models illustrating the relationship between the CPF and the CP.

To clarify the spatial relationship between the CPF and the CP, we examined mEPCs cultured for 5 days after serum starvation (Figure 1A), when multicilia were still undergoing elongation (Delgehyr et al., 2015; Zhao et al., 2021), with 3D-SIM. To determine the relative positioning of different structures along the BB–cilium axis, we mainly compared their side views. Consistent with our previous report (Zhao et al., 2021), the Cep131-decorated CPF was positive for Centrin and positioned further away from the BB distal end marked by Cep162 (Figure 1B, arrowheads), a protein important for the TZ assembly (Wang et al., 2013) and displaying a ring-shaped axial distribution around the BB (Zhao et al., 2021). Immunostaining of Centrin, Cep162, and acetylated tubulin (Ac-tub), a marker of ciliary axoneme, further confirmed the positioning of the CPF in the ciliary central lumen (Figure 1B, arrowheads). Furthermore, the CA visualized through a C2 projection component Hydin or a C1 projection component Spag6 (Teves et al., 2016) appeared to be extended from the CPF (Figure 1C, arrowheads). In contrast, not any CPF was observed at the BBs still in initial stages of ciliogenesis, judged by the lack of Ac-tub-positive axoneme (Figure 1B, arrows) or Hydin/Spag6-labeled CA (Figure 1C, arrows). In such BBs, Centrin localized as a spot at the BB distal end, followed by a slightly expanded cylinder in the centriolar lumen (Figure 1B and C, arrows; Laporte et al., 2022). The Centrin-decorated CPF appeared as an extension from the spot (Figure 1B and C, arrowheads).

Due to the resolution limit of light microscopy, directly staining for MTs (e.g. Ac-tub) could not distinguish CP MTs from peripheral doublets (Figure 1B). We thus used Wdr47, a protein essential for CP formation and predominately distributed at the lattice of central MTs’ minus ends (Liu et al., 2021), to visualize the proximal end of the CP and observed that the proximal end apparently contacted with the CPF (Figure 1D). In some cells, Wdr47 was mainly enriched within a short CP region, possibly due to the relatively short ciliary length (Liu et al., 2021), and the CP proximal end appeared to only slightly overlap with the CPF (Figure 1D, arrowhead in the top panel). Wdr47 was also able to decorate a longer CP region, possibly due to the relatively long ciliary length (Liu et al., 2021), and the CP proximal end tended to display more overlaps with the CPF (Figure 1D, arrowheads in the bottom panel).

Wdr47 is recruited to the lattice of MT minus end by Camsap1, Camsap2, or Camsap3 (Chen et al., 2020; Buijs et al., 2021; Liu et al., 2021), a family of minus-end-binding proteins (Akhmanova and Hoogenraad, 2015; Atherton et al., 2017). As Camsap3 tends to decorate a relatively short region at the CP proximal end (Robinson et al., 2020; Liu et al., 2021; Saito et al., 2021), we expressed green fluorescent protein (GFP)-tagged Camsap3 in mEPCs through lentivirus (Figure 1A). In the mEPCs co-immunostained for Cep131 and Centrin, the end of Camsap3-decorated CP also displayed a different extent of overlap with the CPF (Figure 1E, arrowheads). Furthermore, when Hydin was co-stained with Centrin, the Camsap3-positive CP region nicely bridged the CPF and Hydin-positive CP region (Figure 1F, arrowheads), suggesting that the Camsap3-decorated CP region is devoid of Hydin.

Therefore, the CP proximal end is implanted into the CPF that is assembled at the centriolar distal end in the axonemal central lumen during ciliogenesis (Figure 1G). It becomes increasingly embedded following ciliary growth and the CPF elongation.

### The CPF protrudes from the BB distal end through the TZ into the axonemal central lumen

**Figure 2:**
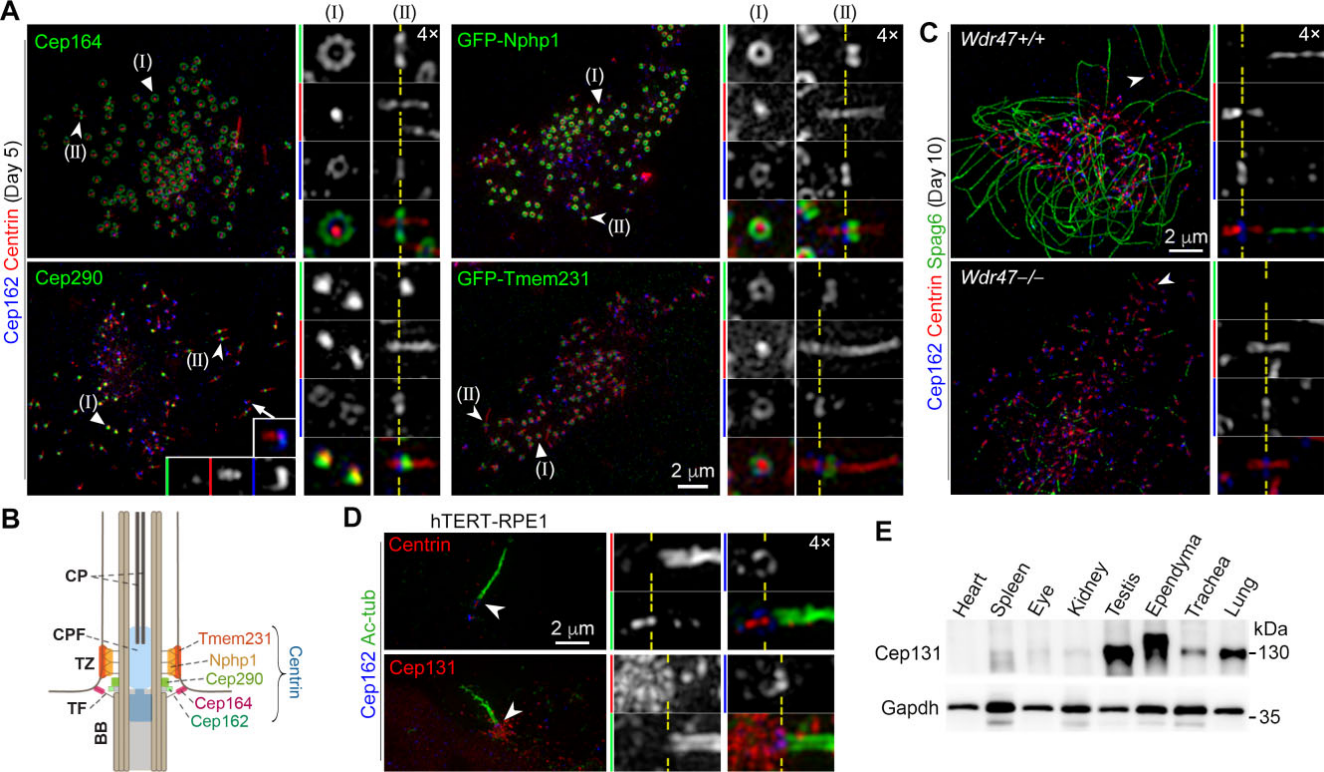
The CPF occupies the center of the TZ and protrudes into the ciliary central lumen independently of the CP. (**A** and **B**) Position of the CPF relative to the centriolar distal end and the TZ. (**A**) mEPCs (uninfected or infected with lentivirus) were immunostained with indicated antibodies. Magnified insets show top views (straight arrowheads) and side views (concave arrowheads) of typical BBs containing the CPF and a representative BB lacking the CPF (arrow) with weak Cep290 localization. Dashed lines indicate the position of the Cep162-marked centriolar distal end. (**B**) The model illustrates the localization of proteins in different subdomains. TF, transition fiber. (**C**) The CPF persists in the absence of the CP. mEPCs were immunostained with the indicated antibodies. Magnified insets show a typical cilium (arrowhead) with (top panels) or without (bottom panels) the CP. (**D**) Primary cilia do not contain a CPF. Ciliated hTERT-RPE1 cells were immunostained and imaged. BBs in magnified insets in **A, C**, and **D** are presented with their proximal-to-distal orientations from left to right. (**E**) Expression profile of Cep131. Tissue lysates were prepared from 8-week-old mice. Gapdh served as a loading control.

Next, we investigated the spatial relationship between the CPF and the TZ. As the precise localization of Cep162 on BBs has not been clearly defined (Wang et al., 2013), we used Cep164, a distal component of transition fibers (Graser et al., 2007; Yang et al., 2015; Garcia-Gonzalo and Reiter, 2017; Shi et al., 2017), to clarify where the CPF started. We observed that the Cep164-labeled ring structure of transition fibers was positioned between the Centrin-labeled CPF and BB. This ring was located in the same plane as the Cep162-marked ring as judged by side views, and both rings encircled the CPF as judged by top views (Figure 2A), confirming that the CPF starts from the central region of the BB distal end (Figure 2B).

TZ proteins occupy distinct regions of the TZ at the ciliary base, where Cep290 forms the base (Figure 2B; Yang et al., 2015). Although Cep290 is recruited to the centriolar distal end at early stages of centriole biogenesis (Tsang et al., 2008; Zhao et al., 2021), it became markedly enriched at a position slightly distal to Cep162 in BBs containing the CPF compared to BBs lacking the CPF (Figure 2A), indicating its enhanced localization upon TZ assembly. Consistent with our previous observation (Zhao et al., 2021), the tip of the CPF generally surpassed the Cep290-marked TZ (Figure 2A and B; Yang et al., 2015; Garcia-Gonzalo and Reiter, 2017; Shi et al., 2017). To clarify whether the CPF protruded out of the Y-link zone, we expressed GFP-tagged Y-link components Nphp1 and Tmem231 in mEPCs (Figure 2A). The CPF also generally protruded out of the TZ labeled by GFP-Nphp1 or GFP-Tmem231 as judged through side views of the BBs (Figure 2A and B). The length of the CPF, however, did not appear to affect the thickness of the Y-link zone (Figure 2A), indicating that the Y-links are assembled independently of the CPF. In top views, 3D-SIM successfully resolved the TZ localization of the Y-link proteins to be a ring slightly larger than that of Cep162. Cep290, which localizes more closely to the MT doublets (Figure 2B; Yang et al., 2015; Garcia-Gonzalo and Reiter, 2017; Shi et al., 2017), appeared only as a speckle (Figure 2A). The CPF was encircled in the ring of the Y-link proteins and largely co-localized with the Cep290 speckle in the corresponding top-view images (Figure 2A), again consistent with its position in the ciliary central lumen (Figure 2B).

### The CPF is assembled independently of the CP and absent in primary cilia

To understand whether the CP was required for CPF assembly, we examined the CPF in *Wdr47-*deficient mEPCs, which lack the CP (Liu et al., 2021). Compared to the long Spag6-positive CA in multicilia of wild-type mEPCs, Spag6 was either completely absent or appeared as one or a few puncta in multicilia of *Wdr47-*deficient mEPCs (Figure 2C), confirming the absence of the CP, though short MT seeds may exist (Liu et al., 2021). The CPF, however, was formed normally in *Wdr47-*deficient mEPCs (Figure 2C). Therefore, the CPF assembly does not require the CP.

Previous expansion microscopy did not indicate an extension of Centrin from the tip of BBs into the ciliary axoneme of RPE1 cells (Laporte et al., 2022), suggesting a lack of the CPF in primary cilia. To verify this, we immunostained primary cilia of RPE1 cells for Cep162, Ac-tub, and Centrin or Cep131. Consistent with a previous report (Laporte et al., 2022), Centrin was only enriched at the distal end and centriolar lumen of the BB (Figure 2D). Similarly, although Cep131 was enriched at the centrosomal area (Hall et al., 2013), it did not localize in the ciliary central lumen (Figure 2D). We further examined tissue distribution of Cep131 and found that Cep131 was highly expressed in mouse testis, ependyma, trachea, and lung (Figure 2E), which contain abundant ‘9 + 2’-type motile cilia (Fliegauf et al., 2007). In contrast, tissues containing only primary cilia, including the heart, spleen, eye, and kidney (Fliegauf et al., 2007), weakly expressed Cep131 (Figure 2E). Therefore, the CPF is a motile cilia-specific structure.

### The CPF is a polarized multicomponent ciliary structure

**Figure 3:**
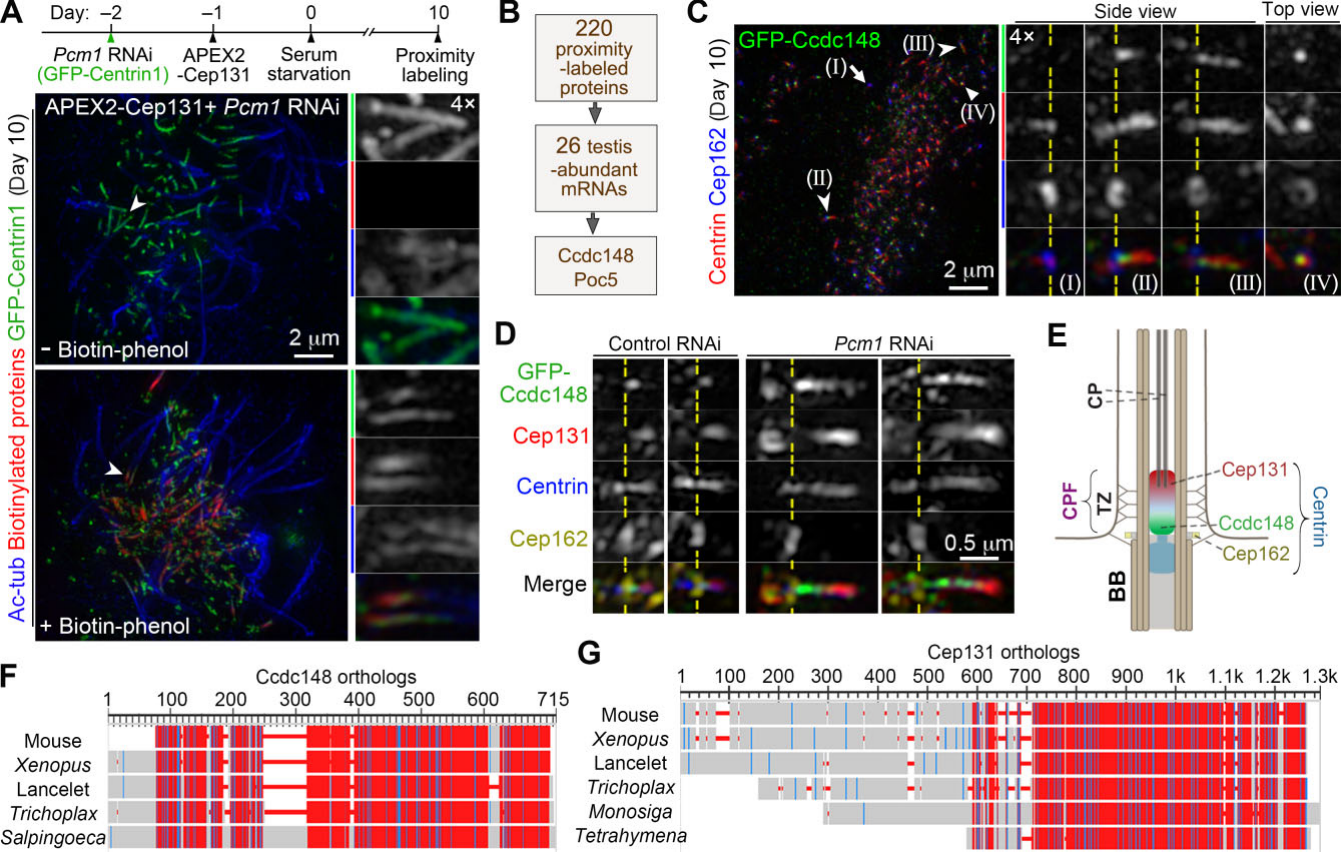
The CPF is a polarized structure consisting of multiple evolutionarily conserved components. (**A**) APEX2-Cep131 biotinylates CPF proteins. mEPCs were either mock-treated (without biotin-phenol) or treated with biotin-phenol and H_2_O_2_. Biotinylated proteins were visualized with dye-labeled streptavidin. GFP-Centrin1 served as a marker for both shRNA expression and the CPF, whereas Ac-tub was used to visualize cilia. (**B**) A flowchart of mass spectrometry data management. From the 26 proteins selectively highly expressed in the testis, Ccdc148 and Poc5 were picked to examine their relationships with the CPF. (**C**) GFP-Ccdc148 is a CPF component. Dashed lines indicate the position of the Cep162-marked centriolar distal end. Arrowheads point to BBs positive for the CPF and GFP-Ccdc148, whereas the arrow denotes a BB lacking the CPF. (**D**) Ccdc148 and Cep131 specify different CPF subdomains. mEPCs expressing GFP-Ccdc148 were transfected with control siRNA or *Pcm1* siRNA. BBs in magnified insets in **A, C**, and **D** are presented with their proximal-to-distal orientations from left to right. (**E**) A model illustrating spatial distributions of the indicated proteins. (**F** and **G**) Conservations among representative Ccdc148 (**F**) or Cep131 (**G**) orthologues from protozoa to mammals. Numbers mark amino acid positions. Red indicates highly conserved positions, and blue indicates lower conservation.

To determine whether the CPF contained components other than Cep131 and Centrin, we performed ascorbic acid peroxidase (APEX)-mediated proximity labeling (Lam et al., 2015) by using APEX2-Cep131 as a bait, followed by shotgun mass spectrometry, to identify potential CPF components, analogous to our previous approach that identified FGM components (Zhao et al., 2021). As we reasoned that longer CPF would enrich more components, we depleted *Pcm1* through small hairpin RNA (shRNA)-mediated RNAi in mEPCs to induce CPF elongation (Zhao et al., 2021) and performed the proximity labeling at Day 10 (Figure 3A). In mEPCs expressing GFP-Centrin1, which served as a marker for both shRNA and the CPF, biotinylated proteins were detected specifically at the CPF after the addition of biotin-phenol (Figure 3A). After removing non-specific proteins by comparing with the hits from the negative control, 220 proteins with peptide spectrum matches ≥5 were identified. After excluding the proteins with known ciliary or FGM localizations, gene expression profiles of the remaining proteins were retrieved from the National Center for Biotechnology Information (NCBI) website, among which 26 exhibited higher expression levels in the testis than in other tissues lacking motile cilia. Considering that proteins encoded by motile cilia-related genes are typically highly expressed in the testis, we regarded these 26 proteins as candidate CPF components (Figure 3B; Supplementary Table S1). Bioinformatic analyses on functions or potential functions of these candidates prompted us to focus on two candidates, Poc5 and Ccdc148 (Figure 3B). Poc5 is a known Centrin-binding protein that targets Centrin to the centriolar lumen (Azimzadeh et al., 2009; Laporte et al., 2022), whereas Ccdc148 is a functionally uncharacterized protein abundant in coiled-coil domains implicated in protein–protein interactions.

By expressing GFP fusions of Ccdc148 and Poc5 in mEPCs through lentiviral infection (Figure 1A), we examined their subcellular localizations. GFP-Ccdc148 displayed a CPF localization, primarily distributed at the base of the CPF in side views of BBs (Figure 3C, concave arrowheads). Sometimes, additional puncta along the CPF were also observed (Figure 3C, concave arrowheads). Top views confirmed its co-localization with Centrin in the ciliary central lumen, encircled by the ring of Cep162 (Figure 3C, straight arrowhead). GFP-Ccdc148 was not detected at BBs lacking the CPF (Figure 3C, arrow), suggesting a dependency on the CPF. In *Pcm1*-depleted mEPCs, the localization of GFP-Ccdc148 along the elongated CPF was enhanced (Figure 3D). Interestingly, in both control and *Pcm1*-depleted mEPCs, GFP-Ccdc148 and Cep131 tended to mark the proximal and distal regions of the CPF, respectively (Figure 3D). These results not only identify Ccdc148 as a new CPF component but also reveal the polarization property of the CPF (Figure 3E).

GFP-Poc5 showed a cylindrical distribution, co-localizing with Centrin in the distal centriolar lumen (Supplementary Figure S1), consistent with previous reports (Azimzadeh et al., 2009; Laporte et al., 2022). Surprisingly, we also observed its presence at the tips of some CPFs (Supplementary Figure S1). Sometimes, a punctum of GFP-Poc5 was observed extending beyond the tip (Supplementary Figure S1). In *Pcm1*-depleted mEPCs, most CPFs were no longer apparently positive for GFP-Poc5, although a few still contained GFP-Poc5 at their tips (Supplementary Figure S1). Poc5 may thus also be a CPF component, which still requires future verifications.

### Both Cep131 and Ccdc148 are conserved from protozoa to mammals

For insights into the evolutionary conservation of the CPF, we searched for taxonomic groups of Ccdc148 and Cep131 in the Protein Database of NCBI (US National Library of Medicine). Their metazoan orthologs could be tracked down to *Trichoplax* of placozoans (Figure 3F and G), the simplest metazoan species (Schierwater et al., 2009; Schierwater and DeSalle, 2018). Nevertheless, no orthologs were identified in nematodes, e.g. *Caenorhabditis elegans*, species containing only immotile sensory cilia (Vincensini et al., 2011), consistent with their roles in motile cilia.

In protists, Ccdc148 and Cep131 homologs were identified in choanoflagellates, such as *Monosiga brevicollis* (Figure 3F and G), the closest known relatives of Metazoa (King et al., 2008; Pinskey et al., 2022). Nevertheless, it appeared that only Cep131 homologs existed in ciliates (*Tetrahymena thermophila*) (Figure 3F and G; Vincensini et al., 2011; Pinskey et al., 2022). The C-terminal region of all these Cep131 homologs was conserved (Figure 3G). In green alga *Chlamydomonas reinhardtii* (Vincensini et al., 2011; Pinskey et al., 2022), only one Cep131 homolog was identified (GenBank accession XP_042921236). It still contained a conserved C-terminal region, but the entire protein (2084 amino acids) was much longer than its protozoan and metazoan homologs. None of these protist proteins, however, was functionally characterized. Taken together, these analyses suggest an evolutionary conservation of the CPF in species containing motile cilia.

### Cep131-deficient mice develop mild hydrocephalus and severe teratozoospermia

**Figure 4:**
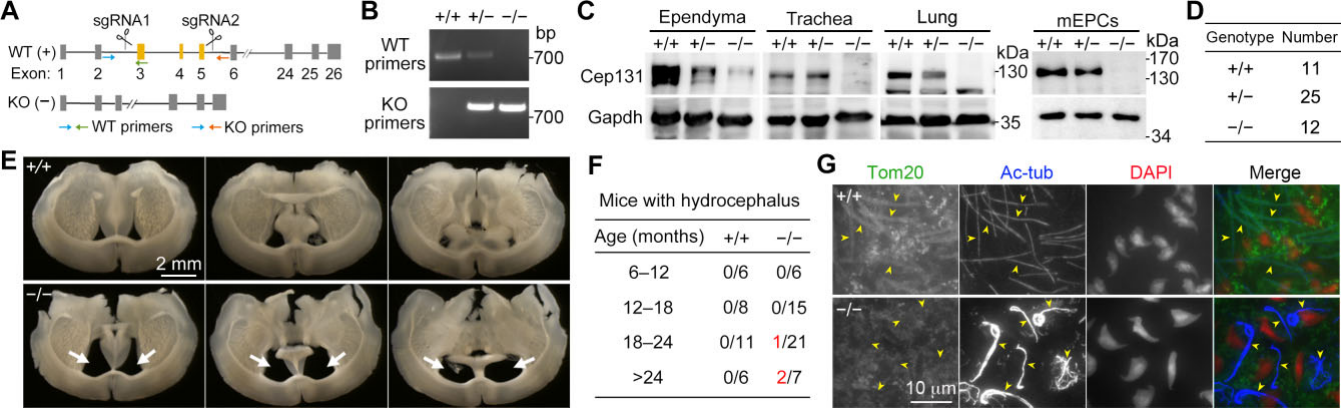
*Cep131* deficiency results in late-onset hydrocephaly and severe teratozoospermia. (**A**) Strategy for generating *Cep131* knockout (KO) mice. (**B**) Representative genotyping results. (**C**) Complete depletion of Cep131 in *Cep131*-deficient mice. Tissue lysates and mEPCs of the indicated genotypes were used to confirm complete depletion of Cep131. (**E** and **F**) Hydrocephaly in aged *Cep131*^−/−^ mice. (**E**) Coronal brain sections were from 22-month-old mice. Arrows indicate enlarged ventricles. (**F**) Frequencies of hydrocephaly in mice of different ages. (**G**) Abnormal flagellar and nuclear morphologies of *Cep131*^−/−^ testicular spermatids or sperms. Spermatids squashed from seminiferous tubules were stained for Tom20, Ac-tub, and nuclei (DAPI) and imaged with a wide-field fluorescent microscope.

To elucidate the physiological roles of the CPF, we generated a *Cep131*-deficient mouse line by genetically deleting a genomic region containing exons 3–5 of *Cep131* through CRISPR/Cas9-mediated gene editing (Figure 4A and B). Immunoblotting confirmed the depletion of Cep131 in the trachea and lung (Figure 4C). Our anti-Cep131 antibody appeared to recognize multiple adjacent bands in mouse ependyma lysates (Figures 2F and 4C). Although the majority of the bands vanished from *Cep131*^−/−^ ependyma lysates, a weak upper band still remained (Figure 4C). We further examined lysates from cultured mEPCs and confirmed the depletion of Cep131 in *Cep131*^−/−^ ependymal cells (Figure 4C). The upper band in the ependyma tissue lysate is thus attributed to antibody cross-reaction.

Previous studies using a gene-trapping mouse line (*Azi1^Gt/Gt^*) revealed that *Cep131* is dispensable for mouse development and biogenesis of both primary cilia and multicilia (Hall et al., 2013). While ∼1/3 of *Azi1^Gt/Gt^* embryos are lost before mid-gestation (Hall et al., 2013), *Cep131*^−/−^ mice were born roughly at the predicted Mendelian ratio based on genotyping results from 6 litters (Figure 4D). Similar to *Azi1^Gt/Gt^* mice (Hall et al., 2013), *Cep131*^−/−^ mice were viable and appeared to be morphologically normal during postnatal development. As defects in the CA of ependymal multicilia probably lead to hydrocephaly in mice (Sapiro et al., 2002; Lechtreck et al., 2008; Liu et al., 2021), we examined serial brain slices from *Cep131*^−/−^ mice of different ages. Interestingly, we observed that 1 out of 21 *Cep131*^−/−^ mice older than 18 months and 2 out of 7 *Cep131*^−/−^ mice older than 24 months had enlarged lateral ventricles (Figure 4E and F). In contrast, 17 wild-type control mice examined randomly had normal ventricles (Figure 4E and F). These results suggest that aged *Cep131*^−/−^ mice tend to develop hydrocephalus.

*Azi1^Gt/Gt^* mice manifest male infertility due to the production of spermatids with both club-shaped nuclei and no or short flagella (Hall et al., 2013). We observed that *Cep131*^−/−^ epididymal sperms were also markedly reduced in number and displayed short or broken flagella compared to those from wild-type animals (Supplementary Figure S2A). When Tom20 was immunostained to label mitochondria (Kanaji et al., 2000), flagella observed in *Cep131*^−/−^ sperm samples mainly consisted of the middle piece (Supplementary Figure S2A), a flagellar region abundant in mitochondria (Vertika et al., 2020). We then examined the morphology of testicular spermatids squeezed out of seminiferous tubules. Compared to wild-type elongated spermatids possessing long axonemes visualized through Ac-tub, axonemes of *Cep131*^−/−^ elongated spermatids tended to be brightly immunostained for Ac-tub, twisted in shape, and evident in signs of disintegration (Figure 4G; Supplementary Figure S2B and C). The middle piece was observed easily in wild-type flagella but rarely in *Cep131*^−/−^ ones (Figure 4G). Furthermore, while wild-type elongated spermatids generally possessed hook-shaped nuclei, the nuclei of *Cep131*^−/−^ elongated spermatids varied from normal hook shape to various aberrant shapes (Figure 4G; Supplementary Figure S2B and C). Therefore, *Cep131* deficiency severely impairs the morphogenesis of spermatids and results in teratozoospermia (Chang et al., 2023) in male mice.

Centrin is degraded in mature mouse sperms (Manandhar et al., 1999). To clarify whether mouse sperm flagella contained the CPF, we performed immunostaining for Centrin and Cep131, respectively, with testicular spermatids. Centrin emerged as a tiny dot at the axonemal proximal end of both *Cep131*^+/+^ and *Cep131*^−/−^ spermatids (Supplementary Figure S2B; Manandhar et al., 1999). Consistent with a previous report (Hall et al., 2013), Cep131 was mainly enriched in a larger spot at the axonemal proximal end of *Cep131*^+/+^ spermatids but the spot disappeared in *Cep131*^−/−^ spermatids (Supplementary Figure S2C). No CPF-like localization was observed for Centrin or Cep131 (Supplementary Figure S2B and C). Therefore, flagella of sperms and elongated spermatids are devoid of the CPF.

### Cep131-deficient ependyma contains multicilia with impaired motility

**Figure 5:**
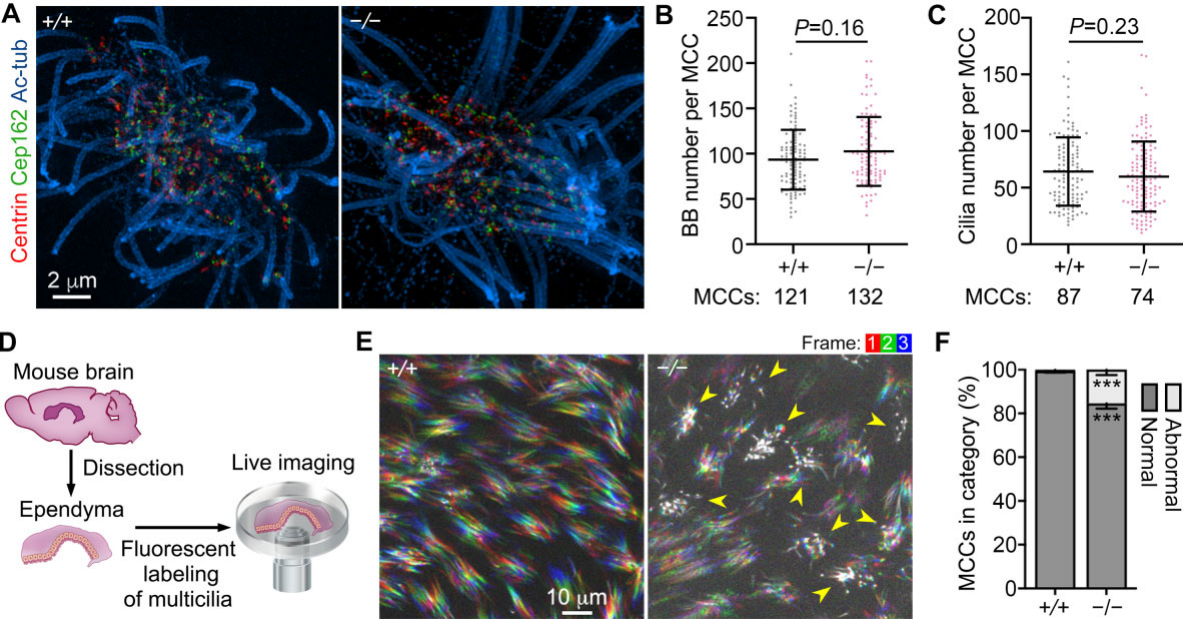
*Cep131* deficiency impairs ciliary motility in the ependyma. (**A**–**C**) Cep131 was dispensable for BB production and ciliogenesis in mEPCs. mEPCs were immunostained with the indicated antibodies and imaged with 3D-SIM. The MCCs of indicated numbers were collected from three independent experiments. Two-tailed Mann–Whitney *U*-test was performed. (**D**) Experimental scheme for imaging ciliary motilities in mouse ependyma tissues. Living brain tissues were dissected from 8-month-old mice, submerged ependyma-side down into a culture medium containing SiR-tubulin, and live imaged at 66 frames per second for 3 sec. (**E** and **F**) *Cep131* deficiency disables motilities of a fraction of cilia. (**E**) The initial three frames from Supplementary Video S1 were overlaid to show cilia beat patterns. Arrowheads point to paralyzed multicilia. MCCs with the majority of multicilia displaying paralyzed motilities were considered ‘abnormal’. Unpaired two-tailed *t*-test, ***P < 0.001.

To clarify whether the depletion of Cep131 impaired ependymal multicilia formation, we cultured mEPCs derived from *Cep131^+/+^* and *Cep131*^−/−^ neonatal mice for 10 days after serum starvation to achieve fully grown multicilia (Delgehyr et al., 2015; Zheng et al., 2019; Liu et al., 2021). The numbers of BBs and cilia were not significantly altered in *Cep131*^−/−^ MCCs (Figure 5A–C), indicating that Cep131 is dispensable for the biogenesis of both BBs and multicilia in ependymal MCCs.

We next sought to examine whether *Cep131*^−/−^ ependymal multicilia displayed abnormal motility. We reasoned that, if the hydrocephalus (Figure 4E) was indeed due to defective ciliary motility, we would be able to detect the abnormality in relatively younger mice. Living ependymal tissues were dissected from 8-month-old littermates and soaked into culture medium (Mirzadeh et al., 2010). Multicilia were labeled with SiR-tubulin, an MT-specific fluorescent dye (Lukinavicius et al., 2014), and live imaged at 15-ms intervals with a spinning disk microscope (Figure 5D). In wild-type ependyma, multicilia within each cell beat in a coordinated back-and-forth manner. Across different cells within the tissue, the cilia predominantly beat in the same direction (Figure 5E; Supplementary Video S1; Ohata and Alvarez-Buylla, 2016). Interestingly, in *Cep131*^−/−^ ependyma, MCCs with paralyzed or slowly waving cilia were observed among MCCs with rapidly beating multicilia. Furthermore, when abnormal cilia were present in the same cell, they tended to beat in different directions (Figure 5E; Supplementary Video S1). Due to the difficulty in scoring MCCs with only a few cilia exhibiting abnormal movement, we scored MCCs with multicilia displaying apparently impaired motility compared to their surrounding cells. We found that ∼15.4% of MCCs in *Cep131*^−/−^ ependyma whereas only 0.6% of MCCs in *Cep131*^+/^*^+^* ependyma exhibited impaired motility (Figure 5F). Therefore, *Cep131* deficiency severely compromises the motility of a substantial portion of ependymal multicilia.

### The CPF requires Cep131 for its formation and functions to uphold the CP

**Figure 6:**
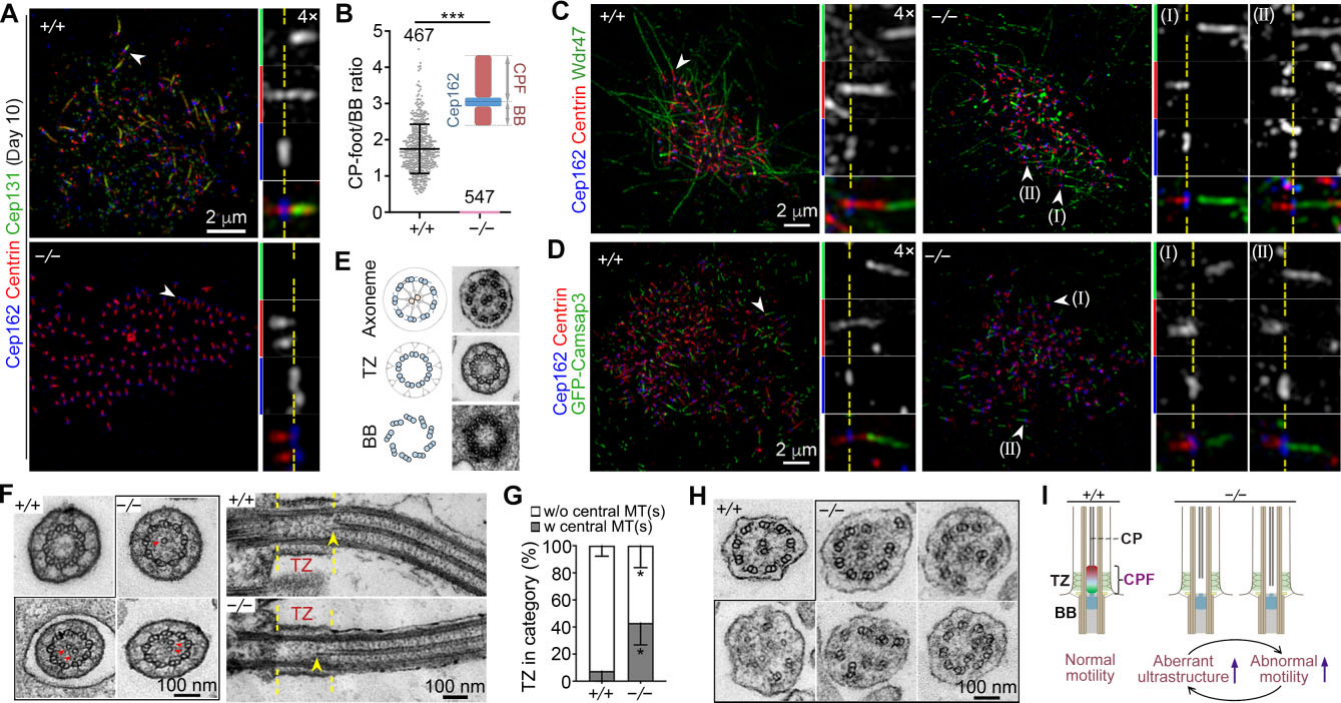
*Cep131* deficiency abolishes the CPF and results in CP and axonemal abnormalities. (**A** and **B**) Complete loss of the CPF in *Cep131*-deficient ependymal multicilia. (**A**) mEPCs were immunostained with the indicated antibodies. (**B**) CPF/BB ratio was measured from side views as illustrated. (**C** and **D**) Loss of the CPF shortens the distance between the centriolar distal end and the CP proximal end. mEPCs (**C**) or mEPCs expressing GFP-Camsap3 (**D**) were immunostained with the indicated antibodies. Representative BBs (arrowheads) in insets in **A, C**, and **D** are presented with their proximal-to-distal orientations from left to right. Dashed lines indicate the position of the Cep162-marked centriolar distal end or the TZ region. (**E**) Representative electron micrographs of 70-nm-thick cross-sections at the indicated positions of ependymal multicilia. Schematics are provided to aid comprehension. (**F**) Representative electron micrographs showing CP penetration into the TZ region of *Cep131*^−/−^ cilia. Red arrowheads in TZ cross-sections point to central MTs. Yellow arrowheads in longitudinal sections point to the CP proximal end. (**G**) *Cep131* deficiency increases the incidence of CP penetration into the TZ region. (**H**) Representative cross-sections showing aberrant axonemal ultrastructure of *Cep131*^−/−^ cilia. (**I**) A model showing that the CPF upholds the CP in cilia of MCCs. *Cep131* deficiency abolishes the CPF, resulting in different extents of CP penetration into the TZ, which impairs the ciliary ultrastructure and ciliary motility.

To clarify whether *Cep131* deficiency could disrupt the CPF, we examined the mEPCs cultured to Day 10 with 3D-SIM. Immunostaining indicated that *Cep131*^−/−^ MCCs were negative for Cep131 (Figure 6A). Furthermore, only the BB localization of Centrin was observed in the MCCs (Figure 6A and B). Therefore, Cep131 is an essential component for CPF formation.

We then investigated whether the loss of the CPF altered CP positioning. In *Cep131*^+/+^ MCCs at Day 10, the CP proximal end marked by Wdr47 or GFP-Camsap3 was usually away from the centriolar distal end marked with Cep162 (Figure 6C and D). However, the CP proximal end in *Cep131*^−/−^ MCCs apparently reached the centriolar distal end in a portion of cilia in SIM images (Figure 6C and D), suggesting penetration of their central MTs into the TZ.

Next, we examined the ciliary ultrastructure in 70-nm-thick ultrathin sections by transmission electron microscopy (Figure 6E). The TZ of wild-type ependymal cilia was usually CP-free in both longitudinal sections and cross-sections, with only 7.4% of the TZ cross-sections containing one or two central MTs (Figure 6F and G). In contrast, 43.0% of *Cep131*^−/−^ TZ cross-sections contained central MTs (Figure 6G). Furthermore, when *Cep131*^−/−^ TZ cross-sections containing a complete CP (i.e. two central MTs), were scored (*n* = 92), 28.3% of the CP was positioned away from the center of peripheral MT doublets (Figure 6F, a representative cross-section), indicating the lack of a holding force that properly positions the CP. Apparent CP invasion into the TZ was also observed in the only clear longitudinal section of *Cep131*^−/−^ cilia (Figure 6F). In addition to the CP mislocalization, we also observed other abnormalities, including disorganized axonemal arrangement and incorrect doublet number, in the cross-sections of *Cep131*^−/−^ cilia (Figure 6H). Quantification of electron micrographs from three littermates per genotype indicated that *Cep131^+/+^* cross-sections exhibited normal ultrastructure (*n* = 90), whereas 25.2% of *Cep131*^−/−^ cross-sections (*n* = 163) contained such abnormalities. Therefore, the CPF functions in upholding the CP in position, and its loss sensitizes axonemes to ultrastructural and beating abnormalities (Figure 6I).

## Discussion

In this study, we demonstrated that the CPF is a polarized substructure in the central lumen of mouse ependymal multicilia (Figure 6I). It is assembled during ciliogenesis from the centriolar distal end, protrudes through the TZ, and terminates in the axonemal central lumen (Figures 1 and 2; Zhao et al., 2021). Following ciliary growth, the CPF also elongates, a process negatively regulated by FGMs (Figure 1; Zhao et al., 2021). In addition to previously reported Cep131 and Centrin (Zhao et al., 2021), we identified Ccdc148 as an additional CPF component from candidates obtained through proximity labeling of APEX2-Cep131 (Figure 3; Supplementary Table S1), though its CPF localization still needs to be validated to exclude potential artifacts associated with protein overexpression. Furthermore, Ccdc148 and Cep131 localize preferentially in proximal and distal subdomains of the CPF, respectively, whereas Centrin distributes along the entire CPF (Figure 3C–E). Therefore, the CPF is not only a multicomponent ciliary substructure but also an intrinsically polarized substructure containing at least two subdomains of distinct molecular compositions. The loss of the CPF upon *Cep131* deficiency (Figure 6) indicated that Cep131 is an essential component. We speculate that Centrin may function as a matrix protein of the CPF, because its depletion in *Chlamydomonas* leads to CP penetration into even the BB (Jarvik and Suhan, 1991; Taillon et al., 1992), whereas Ccdc148 might be involved in initial CPF assembly. Future investigations will be required to verify their functions. In addition, as GFP-Poc5 localizes at the tip of the CPF in some cilia (Supplementary Figure S1), whether Poc5 is a *bona fide* CPF component also awaits future clarifications.

We propose that the CPF serves as a foundation to hold the CA in position both vertically and horizontally for faithful ciliary motility (Figure 6I). Firstly, our SIM studies revealed that CP proximal end initially contacts with the CPF and then becomes increasingly embedded following CPF elongation (Figures 1D–G and 6C, D). Under an electron microscope, the CP is typically observed to terminate at the top of the TZ, but a structure resembling the CPF is invisible (Figure 6F and G; Arima et al., 1984; Liu et al., 2021). Considering the termination of the CPF above the TZ (Figure 1A and B), such a positioning of the CP end is also consistent with our model (Figure 6I). Secondly, when the CPF is abolished through *Cep131* deficiency, the CP penetrates into the TZ, with 28.3% of the penetrated CP positioned away from the center of peripheral MT doublets in cross-sections (Figure 6F), indicating the loss of up-holding forces on the CP end. Notably, although the CP does not penetrate entirely through the TZ in the sole *Cep131*^−/−^ longitudinal electron micrograph (Figure 6F), 3D-SIM results (Figure 6C and D) suggest that the depth of CP penetration varies among *Cep131*^−/−^ cilia. As the thickness of our cross-sections (70 nm) only covered ∼1/3 of the entire TZ (>200 nm), some TZ cross-sections negative for central MTs would actually belong to CP-penetrated TZs. Therefore, the actual incidence of CP penetration is probably higher than the current 43.0% (Figure 6G). Finally, a portion of *Cep131*-deficient ependymal cilia was paralyzed in live imaging (Figure 5) and displayed ultrastructural abnormalities (Figure 6H). We reason that the CP penetration would interfere with the performance of ciliary beat machinery. Aberrant mechanical stresses in the cilia then lead to progressive destruction of normal axonemal ultrastructure, further impairing proper ciliary beat. Such a vicious cycle would thus result in increasingly paralyzed cilia over time (Figure 6I).

Although *Cep131* deficiency seriously paralyzes multicilia in 15.4% of ependymal MCCs in 8-month-old mice (Figure 5E and F), we only observed hydrocephalus in a portion of *Cep131*-deficient mice that were older than 18 months (Figure 4E and F). As the impaired directional flow of cerebrospinal fluid underlies motile cilia dysfunction-induced congenital hydrocephalus (Lee, 2013), we reason that the directional flow of cerebrospinal fluid is maintained in *Cep131*-deficient mice by the majority of normally beating cilia (Figure 5E and F) until the paralyzed cilia increase to a percentage that seriously disturbs the flow in aged mice. Similarly, although tracheal multicilia also contain the CPF (Zhao et al., 2021), neither the previous *Azi1^Gt/Gt^* (Hall et al., 2013) nor our *Cep131*^−/−^ mice displayed obvious ciliopathy-related symptoms in the respiratory system (Fliegauf et al., 2007; Lucas et al., 2020). Future studies are still required to comprehensively understand pathological mechanisms underlying the late-onset hydrocephalus of *Cep131*^−/−^ mice. On the other hand, *Cep131/Azi1* deficiency results in severe teratozoospermia (Figure 4G; Supplementary Figure S2; Hall et al., 2013). As flagella of mouse elongated spermatids or mature sperms do not contain a CPF based on the immunostaining for Centrin and Cep131 (Supplementary Figure S2B and C; Hall et al., 2013), Cep131 probably has a distinct role in spermatogenesis. This is also supported by the concentration of Cep131 in a spot adjacent to the axonemal proximal end (Supplementary Figure S2C; Hall et al., 2013). Alternatively, a CPF might transiently exist during the development from round spermatids to elongated spermatids to facilitate normal spermatogenesis. Future investigations, however, will be required to elucidate molecular roles of Cep131 in spermatogenesis.

The presence of Cep131, Ccdc148, and Centrin homologs from protists to mammals (Figure 3F and G; Aubusson-Fleury et al., 2017) suggests that the CPF is an evolutionarily conserved ciliary substructure. Furthermore, the presence of both Cep131 and Ccdc148 homologs in choanoflagellates (e.g. *M. brevicollis*) but only Cep131 homologs in many protozoa (Figure 3F and G) suggests that, although CPF prototypes emerge in protists, its multi-subdomain structure is initially formalized in the closest known protozoan relatives of Metazoa (King et al., 2008; Pinskey et al., 2022). As Centrin is a common component of both the CPF (Figures 1 and 2; Zhao et al., 2021) and the basal plate (Jarvik and Suhan, 1991; Taillon et al., 1992), we speculate that protist basal plates visualized through electron microscopy (Ringo, 1967; Kennedy and Zimmerman, 1970; Tucker, 1971; Dute and Kung, 1978; Jarvik and Suhan, 1991; Hoog et al., 2014) represent various CPF prototypes. Their selective lack of certain components, e.g. Ccdc148 in ciliates and *Chlamydomonas*, or possession of clade-specific components, e.g. the trypanosome basalin (Dean et al., 2019), may render them strikingly different morphologies under electron microscopes. Certainly, extensive investigations will be needed to verify these speculations.

How the CPF is assembled at a specific location is also an outstanding question that needs to be clarified. Although the CPF length does not impact the thickness of the TZ (Figure 2A), polarized localizations of certain components of the centriolar distal end and the TZ, especially those closely associated with MT doublets, might serve as guidance cues to direct the assembly of different CPF subdomains. The CPF assembly may also be directed by unknown CPF components, which could be identified in the future from the candidate proteins (Supplementary Table S1).

## Materials and methods

### Plasmid constructs and virus

The full-length mouse cDNAs of *Cep131* (NM_009734), *Tmem231* (NM_001033321), *Nphp1* (NM_016902), and *Ccdc148* (NM_001001178) were amplified via polymerase chain reaction (PCR) and subcloned into a lentiviral expression vector, pLV-GFP-C1, to express GFP-fusion proteins. The lentivirus expressing GFP-Camsap3, the adenovirus expressing APEX2-Cep131, and the adenovirus co-expressing *Pcm1* shRNA and GFP-Centrin1 were described previously (Liu et al., 2021; Zhao et al., 2021). All the primers used are listed in Supplementary Table S2. All the constructs were verified by sequencing.

### Mice

Mouse experiments were performed following the ethical guidelines of Shanghai Institute of Biochemistry and Cell Biology, Chinese Academy of Sciences, and approved by the Institutional Animal Care and Use Committee.

*Cep131^+/^^−^* (C57BL/6J) mice were generated using the CRISPR/Cas9 system by Nanjing Biomedical Research Institute of Nanjing University. The genomic region containing exons 3–5 was deleted. *Cep131*^−/−^ mice were generated by inter-crossing heterozygous offspring. Genotyping was performed by PCR with genomic DNA extracted from mouse tails. The forward primer was assigned P1 (5′-CACATTAAGAGTAGTGGTGCACAGC-3′), and the reverse primers were assigned P2 (5′-CTGGTTGACCTGTGTAGTGCTGTT-3′) and P3 (5′-GAAATGCTCTGGAAATGGCC-3′). They are respectively illustrated as cyan, green, and orange arrows in Figure 4A.

### Cell culture, transfection, and viral infection

HEK293T (ATCC, CRL-11268) and HEK293A (Thermo Fisher, R70507) cells were grown in Dulbecco's Modified Eagle Medium (DMEM; Thermo Fisher, C11995500BT) supplemented with 10% fetal bovine serum (FBS; Ausbian, VS500T), 0.3 mg/ml glutamine (Sigma, G8540), 100 U/ml penicillin (Solarbio, P8420), and 100 U/ml streptomycin (Solarbio, S8290). hTERT-RPE1 cells (ATCC, CRL-4000) were grown in DMEM/F12 (Thermo Fisher, C11330500BT) supplemented with 10% FBS, 0.3 mg/ml glutamine, 100 U/ml penicillin, and 100 U/ml streptomycin. Additionally, 10 μg/ml hygromycin B (Thermo Fisher, 10687010) was added to the RPE1 culture medium. Cells were serum-starved for 48 h to induce cilia formation. All cell lines were routinely tested for mycoplasmas.

Cells were transfected at ∼80% confluency using polyethyleneimine (Polysciences, 23966-2) or Lipofectamine 2000 (Thermo Fisher, 11668019) for plasmids and Lipofectamine RNAiMAX (Thermo Fisher, 13778150) for siRNAs. To enhance the knockdown efficiency, control siRNA (5′-TTCTCCGAACGTGTCACGTtt-3′) and *Pcm1*-specific siRNA (5′-GCACCAGGAAUGAAUUUCAtt-3′) were transfected into cells every 3 days (Zhao et al., 2021).

Multiciliated mEPCs were isolated and cultured as described previously (Zhao et al., 2019, 2021; Zheng et al., 2019). Lentiviral and adenoviral particles were produced and used to infect mEPCs as described previously (Zhao et al., 2013, 2019, 2021; Duan et al., 2021; Liu et al., 2021). HEK293T cells were transfected with the lentiviral plasmids, packaging plasmid (Delta 8.9), and envelope plasmid (VSV-G) at a ratio of 5:3:2 for 48 h. To produce adenovirus, the adenoviral expression construct was digested with *Pac*I (New England Biolabs) overnight. The linearized adenoviral expression construct was purified and transfected into HEK293A cells in 6-well plates at 80% confluency. After ∼80% of the cells showed a cytopathic effect, the cells were harvested, and viral particles were released by three freeze-and-thaw (−80°C and 37°C) cycles. The harvested adenoviral particles were added into the culture medium of mEPCs at a 1:200 dilution.

### Immunofluorescent microscopy

Immunofluorescence microscopy of mEPCs was carried out as described (Zhao et al., 2019, 2021). To stain sperm, seminiferous tubules from 2-month-old wild-type and *Cep131*^−/−^ mice were dissected into small pieces of ∼0.5 mm under a stereoscope. These pieces were then squashed on a glass slide with a coverslip and immediately frozen in liquid nitrogen (Kotaja et al., 2004). Sperm samples and cells were fixed with 4% fresh paraformaldehyde (PFA) in phosphate-buffered saline (PBS) for 15 min, extracted with 0.5% Triton X-100 in PBS for 15 min, and blocked with blocking buffer (4% bovine serum albumin in Tris-buffered saline with Tween 20) for 1 h at room temperature. Primary antibodies and secondary antibodies or streptavidin- Alexa Fluor 546 (Thermo Fisher, S11225) were prepared in blocking buffer and applied to cells for 2 h and 1 h, respectively, at room temperature. GFP signals in mEPCs were enhanced by immunostaining using an anti-GFP antibody. All the antibodies used are listed in Supplementary Table S3.

3D-SIM images were captured with DeltaVision OMX SR imaging system (GE Healthcare) equipped with a Plan Apo 60×/1.42 NA oil-immersion objective lens (Olympus) at 125-nm intervals. Raw images were processed for maximum intensity projection with SoftWoRx software.

### Live-cell imaging

As described previously (Mirzadeh et al., 2010; Ohata et al., 2014), after 8-month-old wild-type and *Cep131*^−/−^ mice were euthanized with CO_2_, wholemounts of the lateral wall of lateral ventricles were dissected with Vannas Scissors (Suzhou 66 Vision-Tech, 54140B) in pre-warmed (37°C) dissection solution [117.2 mM NaCl, 5.3 mM KCl, 0.81 mM MgSO_4_, 1.8 mM CaCl_2_, 26.1 mM NaHCO_3_, 1 mM NaH_2_PO_4_·2H_2_O, 5.6 mM glucose, and 25 mM hydroxyethylpiperazine ethane sulfonic acid (HEPES), pH 7.4] under a stereo microscope. The isolated wholemounts of the lateral wall were incubated with 200 nM SiR-Tubulin (Spirochrome, YS-SC002) in dissection solution for 1 h to fluorescently label ependymal multicilia. The SiR-tubulin-labeled ependyma with the ciliated side facing down was transferred to a 35-mm glass-bottom dish (Cellvis, D35-20-1.5-N). The ciliary motility was imaged with a spinning disk confocal microscope (Nikon, Ti2 Microscope) equipped with a Plan Apo 60×/1.42 NA oil-immersion objective (Olympus) and an sCMOS camera (Teledyne Photometrics, Prime 95B). The movies were captured at 15-ms intervals for 3 sec. The laser power was set to 40%, and the exposure time was 14 ms. Kymographs were generated from the time-lapse image sequences with Kymographclear 2.0 of Fiji.

### Electron microscopy

Transmission electron microscopy of ciliary ultrastructure was carried out as described (Zhao et al., 2021). Images were captured at 80 kV using a Tecnai G2 Spirit transmission electron microscope (FEI). To obtain ciliary longitudinal sections, multicilia-containing BBs from one 25-cm^2^ flask of wild-type or *Cep131*^−/−^ mEPCs at Day 10 were purified (Hao et al., 2021). Cells were washed twice with ice-cold PBS and twice with the Ca^2+^-free deciliation buffer (20 mM HEPES, 25 mM KCl, 250 mM sucrose, and 1 mM EDTA, pH 7.5). Then, 3 ml of deciliation buffer containing 0.01% Triton X-100 was added to each flask. The flasks were shaken at 300 rpm for 30 min at 37°C with a horizontal shaker (Zhichu, ZQZY-AS9). The suspension containing released multicilia was collected and centrifuged at 600× *g* for 10 min at 4°C. The multicilia-containing pellet was re-suspended with 1 ml of fresh culture medium and spun onto 12-mm glass coverslips coated with poly-L-lysine (Sigma, P1399) at 4200× *g* for 10 min at 4°C.

### Proximity labeling assay

Biotin-phenol labeling and enrichment were performed as described (Zhao et al., 2021). mEPCs were infected with adenovirus to express *Pcm1* shRNA and APEX2-Cep131 at Day −2 and Day −1, respectively. Cells were serum-starved to induce ciliogenesis for 10 days and then incubated with 500 μM biotin-phenol (Iris Biotech GmbH, LS-3500.1000) at 37°C for 30 min. Subsequently, H_2_O_2_ was added to a final concentration of 1 mM. After 1 min, the biotin-phenol- and H_2_O_2_-containing medium was removed, and cells were washed three times with PBS containing 10 mM sodium ascorbate, 10 mM sodium azide, and 5 mM Trolox to quench the reaction. For immunostaining, cells were fixed and biotinylated proteins were recognized by streptavidin-Alexa Flour 546. For immunoprecipitation experiments, cells were harvested and lysed with radio immunoprecipitation assay (RIPA) buffer supplemented with 1 mM phenylmethanesulfonyl fluoride, 1 mM dithiothreitol (DTT), and the proteinase cocktail (Sigma, 539134). The lysates were cleared by centrifugation at 15000× *g* for 10 min at 4°C. The supernatants were incubated with streptavidin-conjugated agarose beads (Sigma, S1638) for 4 h at 4°C. The beads were washed with RIPA buffer three times. The biotinylated proteins were eluted with 2× protein loading buffer supplemented with 20 mM DTT and 2 mM biotin and boiled for 5 min. The eluted samples were ready for mass spectrometry analysis.

### Brain section

Mice were deeply anesthetized with avertin (360 mg/kg of body weight) by intraperitoneal injection and then transcardially perfused with PBS and 4% PFA in PBS using an injection pump (Smiths Medical, WZS-50F6; 250 ml/h). The brains were dissected and fixed in 4% PFA at 4°C overnight. Fixed brains were then sectioned into 200-μm-thick coronal slices using a Leica VT 1000S vibratome. The brain slices were placed onto 35-mm glass-bottom dishes (Cellvis, D35-20-1.5-N) for imaging. Images were captured with an Olympus SZX16 stereo microscope equipped with an SDF PLAPO 1× PF objective and a DP72 camera.

### Quantification and statistical analysis

Numbers of BBs and cilia were measured from 3D-SIM images. To quantify motility patterns, MCCs with their cilia beating predominantly in a back-and-forth manner were scored as ‘normal’, whereas multiciliated cells containing more than half immotile cilia were considered ‘abnormal’. Kymographs of cilia beating for 3 sec were generated for the calculation of ciliary beat frequency. Quantification results are presented as mean ± standard deviation. Samples that passed the normality test were subjected to a two-tailed unpaired Student's *t*-test using GraphPad Prism software; otherwise, the non-parametric two-tailed Mann–Whitney *U*-test was used to determine statistical significance. Differences were considered significant when *P*-value was <0.05.

## Supplementary Material

mjae031_Supplemental_Files

## Notes

**Author contributions:** X.Z. and X.Y. conceived and conceptualized the research. Q.C. and H.Z. executed major experiments. X.P. contributed to live-cell imaging experiments. C.F. performed western blotting and PCR experiments. B.Q. contributed to the APEX proximity labeling–mass spectrometry experiments. J.G. contributed to bioinformatic analyses. X.Z., X.Y., Q.C., and H.Z. designed experiments, interpreted data, and wrote the paper.
