# Supplementary material for: A polarized multicomponent foundation upholds ciliary central microtubules
Source: J Mol Cell Biol. 2024 Aug 20;16(8):mjae031. doi: 10.1093/jmcb/mjae031 (PMC11781205; doi:10.1093/jmcb/mjae031)
Supplement: mjae031_Supplemental_Files [file mjae031_supplemental_files.zip › JMCB-2023-0516_Supplementary Material.pdf]

Supplementary data

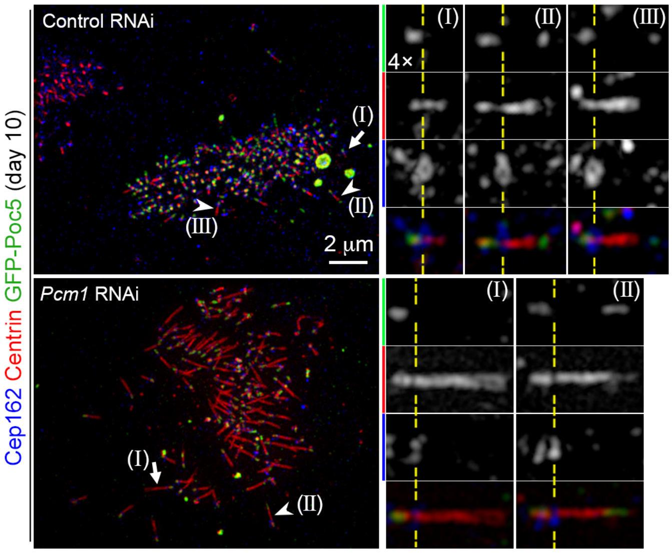

Supplementary Figure S1. GFP-Poc5 displays the CPF-related localization in addition to its known centriolar localization. Cultured mEPCs were infected with lentivirus as illustrated in Figure 1A to express GFP-Poc5. The cells were also transfected with a control or Pcm1-specific siRNA (Pcm-i1) to induce elongated CPF (Zhao et al., 2021). Cep162 served as a marker for the centriolar distal end. GFP-Poc5 was concentrated at or slightly beyond the tip of the CPF marked with Centrin in a portion of cilia. Side views of representative BBs negative (arrows) or positive (arrowheads) for CPF-related GPF-Poc5 are presented in magnified insets, with BB's proximal-to-distal orientation from left to right. Dashed lines mark the position of the centriolar distal end.

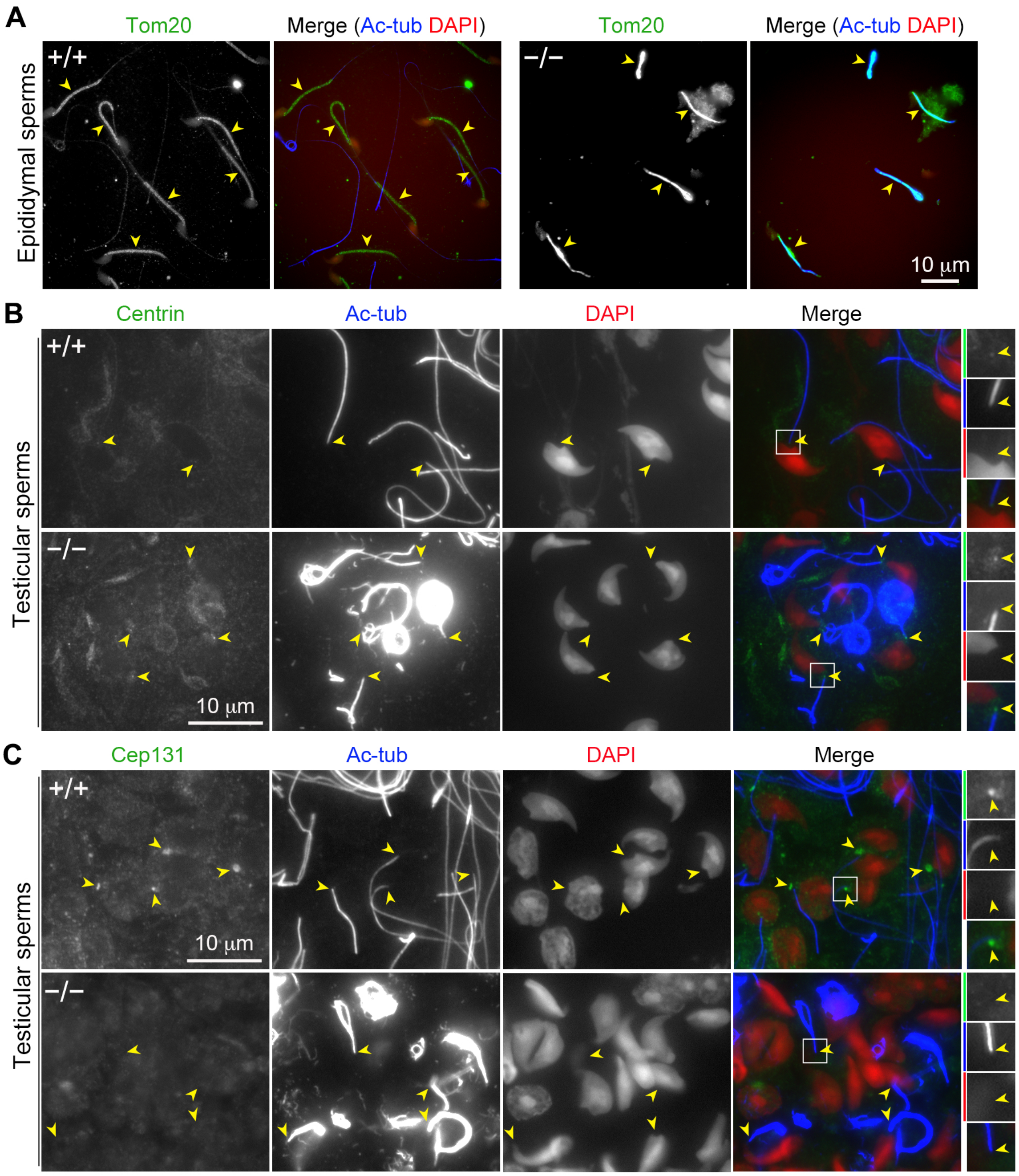

Supplementary Figure S2. Abnormal flagellar and nuclear morphologies of *Cep131*$^{-/-}$ spermatids or sperms. (**A**) *Cep131*$^{-/-}$ sperms mainly displayed short or broken flagella consisting of the middle piece. Sperms were squeezed out of the epididymis of mature male mice. Tom20, acetylated tubulin (Ac-tub), and DAPI label the middle piece (arrowheads), flagellar axoneme, and the nucleus, respectively. (**B, C**) The Flagella of elongated spermatids did not contain a CPF. Testicular spermatids were immunostained for Centrin or Cep131. Ac-tub and DAPI label axonemes and nuclei, respectively. Arrowheads point to positions of Centrin or Cep131 puncta at the bottom end of flagella, except that they denote the bottom end of axonemes for *Cep131*$^{-/-}$ spermatids in (**C**). Framed regions were magnified by 2-fold and are presented as insets to show details. Images were acquired with a wide-field fluorescent microscope.

Supplementary Table S1. List of CPF candidates

Protein Names	Accession	Description	#PSM DMSO	#PSM Biotin-phenol
ASRGL1	Q8C0M9	Isoaspartyl peptidase/L-asparaginase	0	11
**ATP6V1B2**	P62814	V-type proton ATPase subunit B, brain isoform	0	7
**CAPRIN1**	Q60865	Caprin-1	0	5
**CCDC148**	Q6P5U8	Coiled-coil domain-containing protein 148	0	7
**CCDC39**	Q9D5Y1	Coiled-coil domain-containing protein 39	0	5
**CKAP5**	A2AGT5	Cytoskeleton-associated protein 5	0	5
**DDX3Y**	Q62095	ATP-dependent RNA helicase DDX3Y	0	38
**DHX36**	Q8VHK9	ATP-dependent DNA/RNA helicase DHX36	0	6
**DNAJB13**	Q80Y75	DnaJ homolog subfamily B member 13	0	6
**DOCK7**	Q8R1A4	Dedicator of cytokinesis protein 7	0	13
**DZANK1**	Q8C008	Double zinc ribbon and ankyrin repeat-containing protein 1	0	20
**EDC4**	Q3UJB9	Enhancer of mRNA-decapping protein 4	0	8
**FAM161B**	Q8CB59	Protein FAM161B	0	9
**FHAD1**	A6PWD2	Forkhead-associated domain-containing protein 1	0	6
**FXR1**	Q61584	RNA-binding protein FXR1	0	5
**GNL1**	P36916	ARF Guanine-nucleotide exchange factor GNL1	0	6
**LRRC49**	Q91YK0	Leucine-rich repeat-containing protein 49	0	5
**MAPRE3**	Q6PER3	Microtubule-associated protein RP/EB family member 3	0	5
**OPLAH**	Q8K010	5-oxoprolinase	0	11
**PITPNM2**	Q6ZPQ6	Membrane-associated phosphatidylinositol transfer protein 2	0	5
**PNPLA6**	Q3TRM4	Patatin-like phospholipase domain-containing protein 6	0	7
**POC5**	Q9DBS8	Centrosomal protein POC5	0	8
**PSMD2**	Q8VDM4	26S proteasome non-ATPase regulatory subunit 2	0	5
**Ruvbl2**	Q9WTM5	RuvB-like 2	0	9
**TRIM37**	Q6PCX9	E3 ubiquitin-protein ligase TRIM37	0	8

Supplementary Table S2. List of primers used

Plasmid	Insert	GeneBank	Vector	Restriction enzymes for vector digestion	Primer	Sequence (5'->3')
pLV-GFP-Poc5	Poc5	NM_026173	pLV-GFP-C1	XhoI	Poc5-XhoI-F	CCGCTCGAGCTatgtcatctgatgaggacaagtgtt
				EcoRI	Poc5-EcoRI-R	CGGAATTCctagtcaaccacttttatcgactga
pLV-GFP-Tmem231	Tmem231	NM_001033321	pLV-GFP-C1	EcoRI	Tmem231-EcoRI-F	CGGAATTCTatggcgctgtaccatctcttctct
				BamHI	Tmem231-BglII-R	GAAGATCTtcaggataagtgctctttccgtat
pLV-GFP-Nphp1	Nphp1	NM_016902	pLV-GFP-C1	EcoRI	Nphp1-EcoRI-F	CGGAATTCTatgctcgcccggcggccgcgggac
				BamHI	Nphp1-BamHI-R	CGGGATCCttagcctgagttctttcttatttca
pLV-GFP-Ccdc148	Ccdc148	NM_001001178	pLV-GFP-C1	XhoI	Ccdc148-XhoI-F	TCCGGACTCAGATCTCGAGCTatgctgatcaagcaacacaagcagg
				EcoRI	Ccdc148-EcoRI-R	TCGACTGCAGAATTCctagaccttgaacacggtcgattct
pLV-GFP-Camsap3	Camsap3	NM_027171	pLV-GFP-C1	EcoRI	Camsap3-XhoI-F	GACTCAGATCTCGAGGGatggtggaagcggcg
				BamHI	Camsap3-BamHI-R	TGGACTAGTGGATCCctatttgggggtaccgcc
pLV-APEX2-Cep131	Cep131	NM_009734	pLV-APEX2	XhoI	APEX2-Cep131-XhoI-F	GACGCCTCTCGAGCTatgaaaggttcacggacc
				EcoRI	APEX2-Cep131-EcoRI-R	TCGACTGCAGAATTCtcacttagaactcagaga
pYr1.1-APEX2-Cep131	APEX2-Cep131			NheI	pYr1.1-APEX-mCep131-NheI-F	GAACCGTCAGATCCGCTAGCTatggactacaaggatgacgac
				NotI	pYr1.1-APEX-mCep131-NotI-R	TGCAGAGCGGCCGCtcacttagaactcagagat
pYr1.1-GFP-Cetn1-Pcm-i1			pYr1.1-GFP-Cetn1	BsaI	Pcm-i1-F	CACCgcaccaggaatgaatttcaCTCGAGtgaaattcattcctggtgcTTTTTTG
					Pcm-i1-R	AGCTCAAAAAAgcaccaggaatgaatttcaCTCGAGtgaaattcattcctggtgc

Supplementary Table S3. List of antibodies used

Primary antibodies					
**Antigen**	**isotype**	**supplier**	**Cat.#**	**WB**	**IF**
Acetylated tubulin	mouse IgG2b	Sigma-Aldrich	T6793 (6-11B-1)	1: 5000	1: 1000
Centrin	mouse IgG2aκ	Millipore	04-1624 (20H5)		1: 200
CENTRIN1	rabbit IgG	Proteintech	12794-1-AP	1: 1000	1: 200
GAPDH	rabbit IgG	Proteintech	10494-1-AP	1: 5000	
GFP	chicken IgY	Thermo Fisher	A10262		1: 200
Rsph3	rabbit IgG	Proteintech	17603-1-AP		1: 200
CEP131(AZI1)	rabbit IgG	Proteintech	25735-1-AP	1:1000	1: 200
Tom20	rabbit IgG	Proteintech	11802-1-AP		1: 200
mouse Cep131 (full-length)	rabbit IgG	home-made		1:2000	1: 200
mouse Cep290 (2001-2479 aa)	rabbit IgG	home-made		1:1000	1: 200
mouse Cep164 (1-400 aa)	rabbit IgG	home-made			1: 200
mouse Spag6 (full-length)	rabbit IgG	home-made			1: 1000
mouse Hydin (2312-2602 aa)	rabbit IgG	home-made			1: 1000
mouse Wdr47(325aa-597aa)	rabbit IgG	home-made			1: 500
mouse Cep162 (1-300 aa)	guinea pig IgG	home-made			1: 200
**Seconday antibodies**					
**Name**	**Label or Dye**	**supplier**	**Cat.#**	**WB**	**IF**
Goat anti-Rabbit IgG (H+L)	HRP	Thermo Fisher	G-21243	1:5000	
Donkey anti-Mouse IgG (H+L)	Dylinght 405	Jackson ImmunoResearch	715-475-151		1:200
Donkey anti-Rabbit IgG (H+L)	Dylinght 405	Jackson ImmunoResearch	711-475-152		1:200
Donkey anti-Chicken IgY	Dylinght 405	Jackson ImmunoResearch	703-475-155		1:200
Donkey anti-Mouse IgG (H+L)	Alexa Fluor 488	Thermo Fisher	A-21202		1:1000
Donkey anti-Rabbit IgG (H+L)	Alexa Fluor 488	Thermo Fisher	A-21206		1:1000
Goat anti-chicken IgY	Alexa Fluor 488	Thermo Fisher	A-11039		1:1000
Donkey anti-Guinea Pig IgG (H+L)	Alexa Fluor 488	Jackson ImmunoResearch	706-546-148		1:1000
Goat anti-chicken IgY	Alexa Fluor 546	Thermo Fisher	A-11040		1:1000
Donkey anti-Mouse IgG (H+L)	Cy3	Jackson ImmunoResearch	715-165-151		1:1000
Donkey anti-Rabbit IgG (H+L)	Cy3	Jackson ImmunoResearch	711-165-152		1:1000
Donkey anti-Guinea Pig IgG (H+L)	Cy3	Jackson ImmunoResearch	706-165-148		1:1000
Donkey anti-Mouse IgG (H+L)	Alexa Fluor 647	Thermo Fisher	A-31571		1:1000
Donkey anti-Guinea Pig IgG (H+L)	Alexa Fluor 647	Jackson ImmunoResearch	706-605-148		1:1000

Supplementary Materials and Methods

Multiple sequence aligenment

To gain the information on protein conservations among representative Ccdc148 or Cep131orthologues from protozoa to mammals, COBALT (NCBI) was used to generate a graphical overview of multiple protein sequence alignments. The protein sequences of Ccdc148 orthologs (Mouse, AAH62650; Xenopus, XP_004917745; Lancelet, CAH1239120; Trichoplax, RDD42791; Salpingoeca, XP_004990677) and Cep131 orthologs (Mouse, Q62036; Xenopus, NP_001039176; Lancelet, CAH1273747; Trichoplax, RDD45704; Monosiga, XP_001745096; Tetrahymena, XP_001010182) were used for analysis. Numbers mark amino acid positions. Red indicates highly conserved positions and blue indicates lower conservation.

Supplementary video legend

Supplementary Video S1. Ciliary motilities in representative regions of wild-type and *Cep131*-deficient ependymas (related to Fig. 5D-F). Living ependymal tissues from 8-month-old mice were stained with SiR-tubulin to visualize multicilia. Ciliary motilities were live imaged at a fixed z-position at 15 ms intervals for 3 sec with a spinning disc microscope. Image sequences in the first 735 ms are played back at 10 fps. Scale bar, 5 μm.
